# Design, baseline characteristics, and retention of African American light smokers into a randomized trial involving biological data

**DOI:** 10.1186/1745-6215-12-22

**Published:** 2011-01-25

**Authors:** Lisa Sanderson Cox, Babalola Faseru, Matthew S Mayo, Ron Krebill, Tricia S Snow, Carrie A Bronars, Nicole L Nollen, Won S Choi, Kolawole S Okuyemi, Gary A Salzman, Neal L Benowitz, Rachel F Tyndale, Jasjit S Ahluwalia

**Affiliations:** 1 Department of Preventive Medicine and Public Health, University of Kansas Medical Center, Kansas City, KS, USA; 2 Department of Biostatistics, University of Kansas Medical Center, Kansas City, KS, USA; 3 Department of Family Medicine and Community Health, University of Minnesota Medical School, Minneapolis, MN, USA; 4 Program in Health Disparities Research, University of Minnesota Medical School, Minneapolis, MN, USA; 5 University of Missouri-Kansas City School of Medicine, Kansas City, MO, USA; 6 Division of Clinical Pharmacology and Experimental Therapeutics, Department of Medicine and Bioengineering & Therapeutic Sciences, University of California, San Francisco, CA USA; 7 Center for Addiction and Mental Health and Departments of Psychiatry and Pharmacology, University of Toronto, Toronto, ON, Canada; 8 Department of Medicine, University of Minnesota Medical School, Minneapolis, MN, USA; 9 Center for Health Equity, University of Minnesota Medical School, Minnespolis, MN, USA

## Abstract

**Background:**

African Americans experience significant tobacco-related health disparities despite the fact that over half of African American smokers are light smokers (use ≤10 cigarettes per day). African Americans have been under-represented in smoking cessation research, and few studies have evaluated treatment for light smokers. This paper describes the study design, measures, and baseline characteristics from *Kick It at Swope III *(KIS-III), the first treatment study of bupropion for African American light smokers.

**Methods:**

Five hundred forty African American light smokers were randomly assigned to receive bupropion (150mg bid) (n = 270) or placebo (n = 270) for 7 weeks. All participants received written materials and health education counseling. Participants responded to survey items and provided blood samples for evaluation of phenotype and genotype of CYP2A6 and CYP2B6 enzymes involved in nicotine and bupropion metabolism. Primary outcome was cotinine-verified 7-day point prevalence smoking abstinence at Week 26 follow-up.

**Results:**

Of 2,628 individuals screened, 540 were eligible, consented, and randomized to treatment. Participants had a mean age of 46.5 years and 66.1% were women. Participants smoked an average of 8.0 cigarettes per day, had a mean exhaled carbon monoxide of 16.4ppm (range 1-55) and a mean serum cotinine of 275.8ng/ml. The mean Fagerström Test for Nicotine Dependence was 3.2, and 72.2% of participants smoked within 30 minutes of waking. The average number of quit attempts in the past year was 3.7 and 24.2% reported using pharmacotherapy in their most recent quit attempt. Motivation and confidence to quit were high.

**Conclusion:**

KIS-III is the first study designed to examine both nicotine and bupropion metabolism, evaluating CYP2A6 and CYP2B6 phenotype and genotype in conjunction with psychosocial factors, in the context of treatment of African American light smokers. Of 1629 smokers screened for study participation, only 18 (1.1%) were ineligible to participate in the study because they refused blood draws, demonstrating the feasibility of recruiting and enrolling African American light smokers into a clinical treatment trial involving biological data collection and genetic analyses. Future evaluation of individual factors associated with treatment outcome will contribute to advancing tailored tobacco use treatment with the goal of enhancing treatment and reducing health disparities for African American light smokers.

**Trial Registration**

ClinicalTrials.gov: NCT00666978

## Background

Tobacco use remains the leading preventable cause of disease and death in the United States [1]. Differences in patterns of tobacco use, access to treatment, and treatment outcomes between African American and white smokers have been well documented [2-7]. Compared to white smokers, African American smokers consume fewer cigarettes per day and are more likely to be classified as "light" smokers, smoking 10 or fewer cigarettes per day [8-11]. However, African American smokers are more likely to smoke high-tar and mentholated cigarettes [12,13], to inhale more deeply [14], and to have a slower rate of nicotine metabolism [15] and show higher levels of cotinine per cigarette smoked [4,16,17]. Unfortunately, African American smokers are less likely to receive treatment, have poorer treatment outcomes, and suffer a disproportionately greater share of tobacco-related morbidity and mortality [1], making treatment of these smokers a continued public health priority. African American smokers historically have been under-represented in smoking cessation research and few studies have focused on treating light smokers [18,19].

The current paper describes the study design and baseline characteristics from *Kick It at Swope III *(KIS-III), a clinical trial of bupropion and health education counseling for African American light smokers. KIS-III is the third in a series of studies aimed at enhancing tobacco use treatment for African American smokers. The first KIS-I trial [20] was a double-blind, placebo-controlled trial that demonstrated the efficacy of sustained release bupropion in African American moderate to heavy smokers, those who smoked 10 or more cigarettes per day (cpd). Because over 50% of African American smokers are light smokers, the second KIS trial (KIS-II) focused on treating light smokers (1-10 cpd) and evaluated nicotine gum compared to placebo in combination with either motivational interviewing (MI) or health education (HE) counseling using a 2x2 factorial design [21,22]. Findings from KIS-II showed no benefit of nicotine gum, but demonstrated a doubling in rates of abstinence for smokers who received HE compared to MI counseling [22]. The lack of response to nicotine replacement therapy may have been due to under-dosing related to low dose of drug used (2mg rather than 4mg), insufficient frequency of dosing, or poor adherence. For these reasons, a non-nicotine medication was considered for the present trial of African American light smokers. Of note, we explored more completely the issues of biomarkers and found neither carbon monoxide nor cotinine were strongly associated with self-reported smoking level in this light smoking population [4]. We also found that abstinence was influenced by genetically variable rates of nicotine metabolism [15].

The present KIS-III study was designed to build upon KIS-I and KIS-II findings. Because bupropion was found to be effective in African American moderate to heavy smokers, KIS-III was designed to extend this evaluation within African American light smokers, in combination with effective HE counseling. Based on prior research, bupropion treatment is expected to reduce the rewarding effects of smoking and aid withdrawal reduction for smokers independent of smoking level. In addition, we continued investigation of genetic and pharmacokinetic influences on cessation in KIS-III.

Because genetic, sociocultural, and pharmacological determinants of smoking vary across racial and ethnic groups and may impact tobacco use treatment, empirical evaluation of these factors in the context of smoking cessation intervention has been recommended [15,16,23]. The consistent disparity of finding higher cotinine levels per cigarette in African American smokers is likely due to greater intake of nicotine per cigarette smoked combined with slower nicotine metabolism [4,17]. The enzyme CYP2A6 [24,25] and genetic variation in CYP2A6 is associated with multiple smoking behaviors including cessation, as well as tobacco-related disease risk [25-28]. Genetic variation in the *CYP2B6 *gene, coding the enzyme responsible for bupropion metabolism, is also associated with smoking cessation in the context of both bupropion and placebo treatment [29-31]. KIS-III was designed to extend previous studies of African American smokers by incorporating biological evaluation of both nicotine and bupropion metabolism, and the genetic variation in the main metabolic enzymes CYP2A6 and CYP2B6, in addition to other smoking and psychosocial factors, within this clinical trial of light smokers. This paper describes the study design, baseline characteristics and retention of African-American light smokers into a smoking cessation treatment trial that involves biological data collection.

## Methods

### Study Design

This is a randomized, placebo-controlled study with the primary aim of evaluating the efficacy of sustained release bupropion in combination with health education (HE) counseling for smoking cessation among urban African-American light smokers. Five hundred and forty African-American light smokers recruited from the Kansas City metropolitan area were randomly assigned to an active bupropion and health education (HE) counseling (Tx) condition (n = 270) or to a placebo and HE comparison (C) condition (n = 270). The primary outcome was 7-day point prevalence smoking abstinence at 6 months confirmed with salivary cotinine. The study was conducted at an urban community-based clinic that serves predominantly low-income African-American patients. A Community Advisory Board (CAB) composed of African-American community members assisted in the implementation of the study. The study procedures were approved and monitored by the University of Kansas Medical Center Human Subjects Committee.

### Study Sample

#### Participant Recruitment

Recruitment started in December 2007 and ended in October 2009. The final 6-month follow-up was completed in May 2010. Participants were recruited through clinic- and community-based efforts. Clinic-based efforts included use of fliers, posters, physician letters, pharmacy inserts and lobby recruitment at the primary study site, Swope Health Services in Kansas City, two Swope affiliate clinics, and two regional hospitals (the University of Kansas Medical Center and Truman Medical Center). Community-based efforts included advertisement through radio, television, and newspapers, informative presentations at health fairs and libraries, local businesses and religious organizations, and website communication. Participants received referral cards to distribute to friends who smoked. Newsletters providing current project information, recruitment and success stories, and tips for quitting and relapse prevention were also mailed to participants to share with other smokers.

#### Eligibility and Screening

Interested individuals contacted us by telephone or in-person. Study staff provided smokers with detailed information about the study and offered screening for eligibility. To be eligible, individuals self-identified as African-American, were age 18 or older, interested in stopping smoking, smoked ≤10cpd for ≥ 2 years, smoked on ≥25 days in the past month, and were willing to attend 4 clinic visits over the course of 6 months. They must have smoked for at least 3 years, have a home address and a functioning telephone number. Exclusion criteria included current use of bupropion; use of psychoactive medications; use of nicotine replacement therapy, fluoxetine, clonidine, buspirone, or doxepin in the past 30 days; history of alcohol or substance abuse within the past year; current drinking of 14 or more alcoholic drinks per week and/or binge drinking (5 or more drinks on one occasion) 2 or more times in the past month; history of seizures or head trauma; history of bulimia or anorexia nervosa; pregnant (verified by over the counter pregnancy test kit for women of child-bearing age only) or contemplating pregnancy; breast feeding; myocardial infarction in the past 30 days; use of other forms of tobacco in the past 30 days; reported use of opiates, cocaine, or stimulants; diabetes treated with oral hypoglycemics or insulin; planning to move from the Kansas City metro area in the next 12 months; and having another smoker in the household enrolled in the study.

#### Consent and Randomization

Study staff reviewed procedures with individuals who were eligible to participate in the study and administered written informed consent to these individuals at the baseline visit. As part of the consent process, potential participants were informed about mandatory collection of blood samples for DNA analysis and procedures for collecting and handling of the samples. Study staff explained the rationale for the genetic analysis, i.e. to understand individual differences in nicotine and bupropion metabolism and smoking behavior among African-American smokers, and how these factors influenced the likelihood of successfully stopping smoking. The consent form specified that (a) analysis of DNA ''cannot and will not be used for my own benefit and no results, even if unfavorable, will be forwarded to me,'' (b) DNA would be stored for a period of no less than 10 years, (c) participants could request at any time that their DNA be destroyed, (d) while results of the study may be published, no identifying information would be included in publications and confidentiality would be maintained, and (e) blood samples would not be used to develop a commercial product. Participants responded yes or no to the question, ''I give permission for my DNA to be used in future studies about smoking.'' Participants also were asked for permission to be contacted for future studies that might require additional information. A computer generated random numbers table was used to randomize participants into an active bupropion and health education (HE) counseling (Tx) condition or to a placebo and HE counseling (C) condition. Both participants and investigators were blinded to the pharmacotherapy condition.

### Intervention

Participants received pharmacotherapy treatment (bupropion or placebo) for 7 weeks, health education (HE) counseling through Week 16, and were followed through Week 26. All participants received *Kick It at Swope: Stop Smoking Guide, *a culturally sensitive smoking cessation guide developed for African American light smokers and used previously [22].

#### Bupropion

At baseline (Week 0), a research assistant gave each participant a 7-week supply of bupropion SR 150 mg (150 mg daily for 3 days, then 150 mg twice daily) or placebo. All participants received an instruction sheet on effective use of bupropion. A scheduled quit date was planned to follow 7 days of pharmacotherapy use. Adverse events were assessed at Weeks 1, 3, 5, 7, and 16. Serum levels of bupropion were drawn at Week 3 for measurement of concentrations of bupropion and metabolites.

#### Counseling

Health Education (HE) counseling is an education-based intervention that incorporates recommendations from the current tobacco treatment guidelines: giving clear advice to stop smoking, providing assistance with quitting, and arranging follow-up [18]. In this study, HE counseling included providing information about the risks of continued smoking and the benefits of quitting, developing a quit plan, outlining a concrete quit day preparation plan, discussing strategies for successful quitting, building social support, reducing stress, recognizing and managing withdrawal and craving, overcoming barriers to abstinence, and using pharmacotherapy. Table 1 provides a description of the six HE sessions provided to participants in both arms of the study in person at Weeks 0, 1, 3, 7, and by phone at Weeks 5 and 16. Study staff used semi-structured scripts for HE sessions to incorporate counseling with use of the *Kick It at Swope: Stop Smoking Guide*, but worked to tailor information to the individual while assisting participants in developing personalized stop smoking plans. HE sessions lasted, on average, 15-20 minutes.

**Table 1:** Overview of Health Education Counseling Sessions

Session Number	Goal	Topic
Randomization, Week 0 (30 minutes)	Establish rapport with participant-emphasizing willingness to help them quit and encouraging their motivation/confidence to quit smoking.	AA tobacco use, health risks, benefits of quitting, learning from past quit attempts; and developing a plan for quit day. Instructions of medication use as well as Identifying triggers and managing withdrawal were also reviewed

Week 1 (15 minutes)	Reinforce quit day plan, address medication use, identify concerns, barriers, and strategies for success	**If Quit**: Rewarding yourself, recovering from slip, review medication use or ending medication (week 7), managing stress, alternatives to smoking, identify barriers, and living smoke-free
	
Week 3 (20 minutes)	Reinforce and encourage abstinence efforts. Identify concerns, barriers, and strategies for successes	
		
Week 5 (15 minutes)		
		
Week 7 (20 minutes)		
		
Week 16 (15 minutes)		**If still smoking**: Review reasons for not quitting, review reasons for quitting, discuss specific problems that lead to relapse, and attempt to set a new quit plan

Weeks 0, 3, 7 conducted in person. Weeks 1, 5, 16 conducted via telephone.

#### Written materials

At baseline, study staff reviewed the *Kick It at Swope Stop Smoking Guide *with participants. The 36-page guide includes information about health consequences of tobacco use and benefits of quitting, disproportionate effects of tobacco on African-American smokers, menthol, light smoking and related risks. Specific strategies to promote abstinence included making a quit plan, using medication, obtaining social support, managing withdrawal and craving, exercise and healthy eating, coping with a lapse and relapse prevention.

##### Retention

Prior to each study visit, study staff completed telephone calls and sent postcard to all participants of scheduled appointments. For any missed session, participants received up to 6 telephone calls to facilitate rescheduling. Study staff gave each participant a $20 gift card for completing each in person visit (Weeks 0, 3, 7) and a $40 card for completing follow-up at Week 26, in appreciation of participant time and effort. Remuneration was based on session attendance and not on smoking status. Staff also distributed small tokens (e.g., tote bag, t-shirt, museum passes) to participants for in person and telephone sessions completed throughout the study.

### Measures

Table 2 provides an overview of assessments conducted over the course of KIS-III. Study staff verbally administered all self-report measures.

**Table 2:** KIS III Assessments

Assessment	# items	Ranges of Scores	Eligibility	Randomization Week 0	Week 1	Week 3	Week 5	Week 7	Week 16	Week 26
Blood draw				X		X				

Salivary cotinine										X

Demographics	7		X	X						

Cigarette Data	5			X						

Other Tobacco Use	1		X	X				X		X

Smoking Status (cpd)	1		X	X	X	X		X		X

Timeline Follow-back (TLFB)	7			X		X		X		X

Smoking History	4			X						

Quitting History	4			X						

Weight Concerns:										

Weight Concerns Scale,	6	1-6								
Weight Self Efficacy Scale,	6	1-10		X						
Adapted Myers Weight Concerns	2	0-11								

Home Smoking Restrictions	5			X				X		

Work Place Smoking Restrictions	4			X						

Motivation to Quit	1	1-10		X						

Confidence to Quit	1	1-10		X						

Nicotine Dependence (FTND)	6	0-10		X						

Wisconsin Inventory of Smoking Dependence Motives (WISDM-30)	30	10-70		X						

Minnesota Withdrawal Scale (MNWS)	8	0-32		X		X		X		X

Craving (QSU-Brief)	10	1-7		X		X		X		X

Positive and Negative Affect (PANAS)	20	10-50		X		X		X		X

Depression:										

CES-Short Depression Scale (CESD-10),	10	0-30								
2-item Patient Health Questionnaire (PHQ-2)	2	0-6		X		X		X		X

Stress (PSS-4)	4	0-16		X						

Social Support (ISEL-12)	12	12-48		X						

Impulsivity	5	5-20		X						

Racial Discrimination [45]	12	0-9		X						
		0-45								

Racial/Ethnic Identity	8			X						

Approximate duration of survey (in minutes)			10	45	5	15	5	15	5	10

#### Demographic measures

Demographic information during the baseline assessment was collected using standardized questionnaires. This information included age, gender, marital status, income, employment status and education. Height and weight were measured to calculate body mass index.

#### Smoking behavior measures

Baseline assessment of smoking history included current number of cigarettes smoked per day (CPD), type of cigarette smoked (menthol or non-menthol), age when first smoked, age when started smoking regularly, quitting and relapse history, reason for most recent relapse, and implementation of home smoking restrictions. Timeline follow back [32] was employed to assess individual patterns of smoking over the past week. Participants rated motivation and confidence for quitting on a ten point scale, with higher scores reflecting greater motivation or confidence. Nicotine dependence was measured using the Fagerström Test of Nicotine Dependence (FTND) [33] and the Wisconsin Inventory of Smoking Dependence Motives, 30 items (WISDM-30) [34-36]. Nicotine withdrawal in the past 24-hours was assessed using the Minnesota Nicotine Withdrawal Scale (MNWS) [37]. The brief version of the Questionnaire for Smoking Urges (QSU-brief) assessed craving to smoke [38].

#### Psychosocial measures

The Center for Epidemiologic Studies Short Depression Scale (CESD-10) assessed distress associated with depressive symptoms [39,40]. The 2-item Patient Health Questionnaire (PHQ-2) was also used to identify primary symptoms of depression over the past two weeks [41]. The Perceived Stress Scale, 4 items (PSS-4) assessed self-appraised stress experienced in the past month [42]. The 12-item Interpersonal Support Evaluation List (ISEL-12) assessed global level of social support [43]. The Positive and Negative Affect Scales (PANAS) were administered to measure positive (e.g., alert, enthusiastic) and negative (e.g., anger, fear) affective states [44]. We used the Experiences of Discrimination (EOD) scale to assess the frequency of self-reported discrimination because of race, ethnicity or color [45,46]. The Reward Responsiveness Scale of the Behavioral Inhibition System/Behavioral Activation Scale (BIS/BAS) assessed behavioral inhibition, behavioral activation, and affective responses to impending reward and punishment [47]. Smoking-related weight concerns and confidence to maintain current weight after quitting were assessed using the Meyers Weight Concerns Scale [48], a modified version of the Weight Concerns Scale [49], and the Weight Efficacy After Quitting scale (WEAQ) [49].

### Biochemical measures

#### Nicotine metabolism phenotype (3HC/COT ratio) and genotype

Cytochrome P450 (CYP) 2A6 is the major enzyme responsible for metabolizing nicotine into cotinine (COT) and the further metabolism of COT to trans-3'-hydroxycotinine (3HC) [50,51]. Cotinine, in turn, is metabolized into trans-3'-hydroxycotinine (3HC) also by CYP2A6 [51]. The half-life of nicotine (2 hours) is relatively short compared to cotinine (16 hours) [52]. Trans-3'-hydroxycotinine (3HC) is formation dependent, resulting in a half life that is the same as its parent cotinine (16 hours). Therefore, the ratio of 3HC to cotinine is fairly constant over time [53]. The plasma 3HC/COT ratio is highly correlated with total nicotine clearance and CYP2A6 activity making it a useful biomarker of the rate of nicotine metabolism [54]. Both the 3HC/COT ratio and/or *CYP2A6 *genotype have been associated with the number of cigarettes smoked per day and the likelihood of quitting [27,55,56], although disparate findings suggest further study is needed particularly among different racial/ethnic groups [57-60].

#### Bupropion metabolism phenotype and genotype

Bupropion acts, in part, by inhibiting norepinephrine and dopamine reuptake [61]. Bupropion has three principal metabolites: hydroxybupropion, threohydrobupropion, and erythrohydrobupropion [62]. The mean elimination half-lives for these metabolites are estimated to be 20 hours for hydroxybupropion, 37 hours for threohydrobupropion, and 33 hours for erythrohydrobupropion. These metabolites are known to be pharmacologically active, although their relative activity is not well established [29]. Following 150mg of sustained-release bupropion every 12 hours, steady state plasma concentrations for bupropion and metabolites are reached within 8 days.

CYP2B6 metabolizes bupropion to its main metabolite 6-hydroxybupropion [63,64]. Genetic variation of CYP2B6 alters enzyme activity [30,65-68], and genetic variation in CYP2B6 has been associated with differences in smoking cessation outcomes [30].

#### Blood and Serum Collection

Blood samples were collected at two time points: Weeks 0 and 3. Samples were collected at Week 0 for evaluation of nicotine metabolism phenotype (3HC/COT, derived from nicotine from smoking) and both CYP2A6 and CYP2B6 genotype. Participants smoked one cigarette of their usual brand: after 30 minutes, 20 ml of blood was drawn into polypropylene containers- B-D vacutainer containing 100 USP units Lithium Heparin, mixed by inversion and centrifuged at room temperature for 10-15 minutes. Plasma was extracted and stored at -20° C. Following completion of data collection, plasma samples will be thawed at room temperature and then assayed by solid phase extraction followed by LC/MS/MS [69] to evaluate nicotine, COT and 3HC using standard procedures [70,71]. For genotyping, 20 mls of blood was collected into ACD tubes, mixed and then transferred samples to 20 ml plastic scintillation vials which were frozen at -20°C. *CYP2A6 *and *CYP2B6 *genotyping will use published gene and allele-specific polymerase chain reaction assays [4,31,51].

Week 3 was selected as the time point for the analysis of bupropion metabolism phenotype, as participants using active bupropion (n=270) will have 1) reached steady state levels of bupropion, and 2) passed at least 7 days from their Quit Date, allowing us to evaluate bupropion metabolism in relation to cotinine-confirmed 7-day point prevalence abstinence. Because participants and study staff were blinded to treatment condition, blood was collected from all participants. Participants reported the date and time of their most recent medication dose and their daily use of study medication during the previous 7 days, and the information were recorded in relation to the date and time of blood draw. Following completion of all participant data collection, follow-up, and subsequent unblinding of randomization, phenotype analysis will be conducted for samples of participants from the active bupropion arm of the study only.

#### Salivary cotinine validation of smoking abstinence

The primary endpoint of this study is biochemically verified 7-day point prevalence smoking abstinence defined as no cigarettes (not even a puff) in the previous 7 days at Week 26, validated using salivary cotinine collected at Week 26. This method is consistent with recommended guidelines [72,73]. We used the cut-point of 15ng/ml cotinine to differentiate smokers from nonsmokers [73-75]. Salivary cotinine analysis was conducted using gas chromatography technique as described elsewhere [76].

### Data analysis

Within this paper, we calculated descriptive summaries of baseline (Week 0) demographic, psychosocial characteristics and smoking history of the participants using frequencies and percentages for categorical variables and means and standard deviations for quantitative variables. Phenotyping, genotyping, and evaluation of abstinence outcomes will be presented in future manuscripts.

## Results

**Figure 1:**
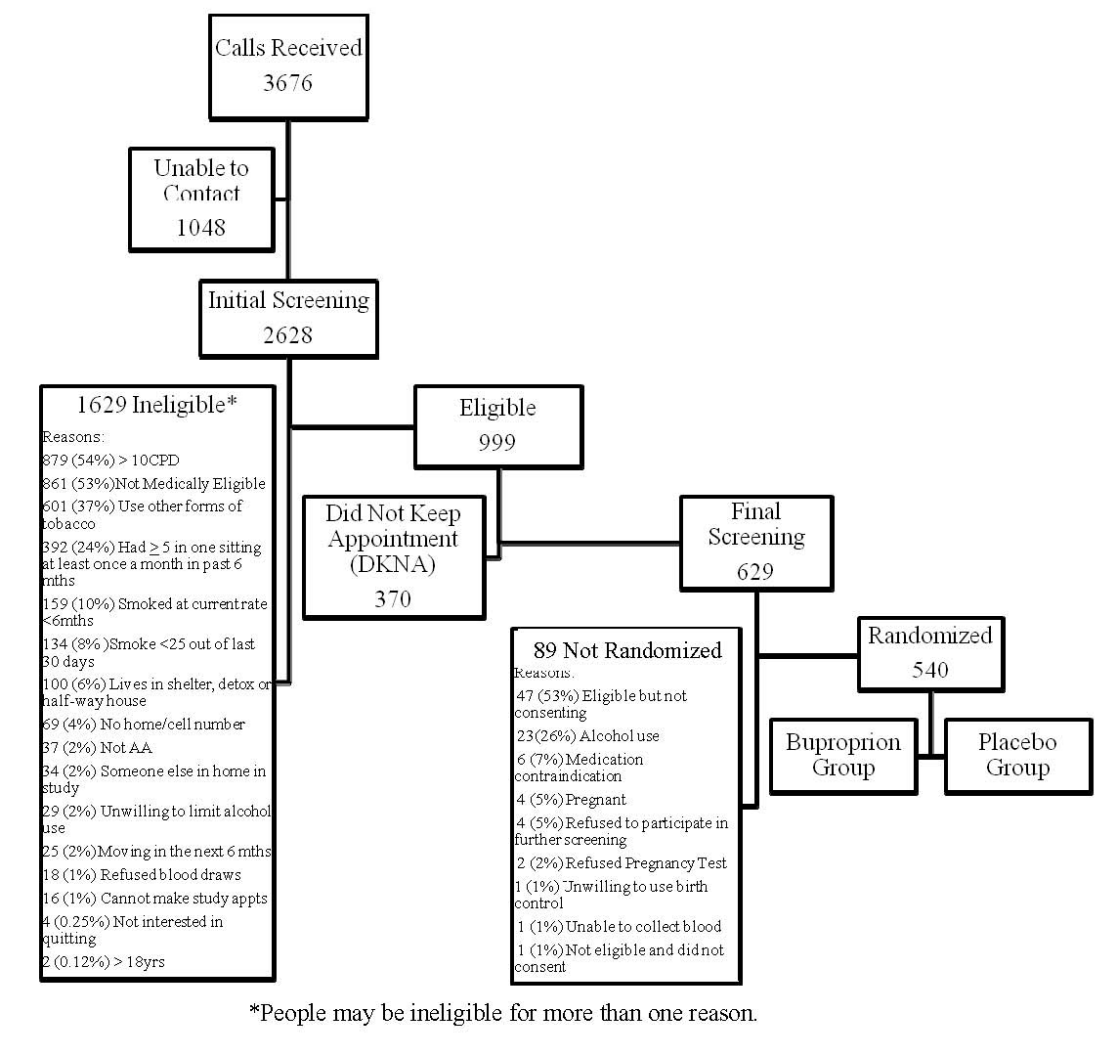
**Screening and enrollment of study participants**.

An overview of screening and enrollment for KIS-III is provided in Figure 1. Of 2,628 individuals who expressed interest in study participation and were screened for eligibility, 999 (38%) were phone-eligible and were scheduled for in-person screening. Radio was the most common source of information about the study (29%), followed by recruitment through the Swope clinic (20%), television (17%), word of mouth (13%), and Truman Medical Center (9%). Most of those screened (70%) were ineligible for study participation with the primary reasons for ineligibility being smoking >10 CPD, not medically eligible, use of other forms of tobacco, and binge drinking within the past 6 months. Of smokers who passed phone eligibility, one third (370) did not keep the final eligibility study appointment. Of those who attended final eligibility screening (629), 89 (14%) were not eligible for randomization. Five hundred and forty (86% of those attending final eligibility screening) were eligible, consented, and randomized to the bupropion or placebo treatment group.

**Figure 2:**
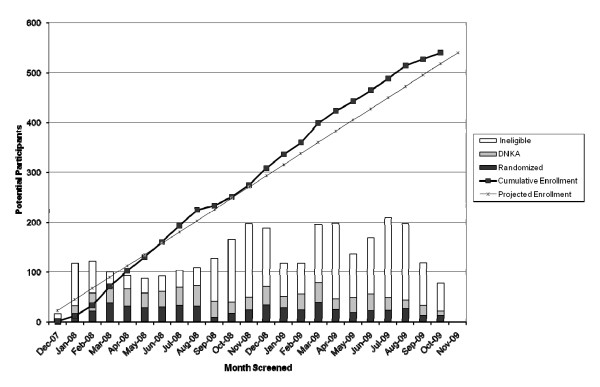
**Cumulative Enrollment and Number of Randomized, Those Who Did Not Keep Their Appointment (DNKA), and Ineligible Participants**.

Participant randomization by month (from December 2007 to October 2009) is presented in Figure 2. The bar graph represents monthly numbers of individuals screened, those ineligible, those who did not keep appointment and participants randomized, while the line graph reflects cumulative enrolment over the study period. Participant enrollment was completed 7 weeks ahead of the projected schedule.

Table 3 presents baseline characteristics of KIS-III study participants. Participants had a mean age of 46.5 years, the majority, (66.1%) were women, one in three were married or living with a partner, and over 84% had obtained a high school degree. Almost two in three earned less than $1,800 monthly. Participants smoked an average of 8.0 cigarettes per day, had a mean exhaled carbon monoxide of 16.4ppm with a range of (1-55), a mean cotinine of 275.8ng/ml. Mean Body Mass Index (BMI) was 31.1. Over 72% smoked within 30 minutes of waking with a mean FTND dependence score of 3.2 with a range of (0-7). Most (83.7%) reported smoking menthol cigarettes. Only 24.4% reported living in a smoke-free household. The average number of quit attempts in the past year was 3.7 and 24.2% reported using pharmacotherapy during their most recent quit attempts. Motivation and confidence to quit were high. These demographic and smoking history characteristics are similar to African American light smokers from our previous KIS-II trial [22].

**Table 3:** Participant Characteristics

	Summary statistics	Sample size (n)
**Demographic Variables**		

Age, mean [SD] yr	46.5 (11.3)	540

Women, n (%)	357 (66.1)	540

Married or living with partner, n (%)	166 (30.8)	539

Monthly family income < $1800, n (%)	327 (60.7)	539

Education ≥ High school, n (%)	454 (84.2)	539

Weight, mean [SD]	195.5 (52.9)	539

BMI, mean [SD]	31.1 (7.9)	539

**Psychosocial Variables**		

Depression (CESD-10), mean [SD]	7.7 (5.2)	539

Stress (PSS-4), mean [SD]	5.2 (3.2)	539

**Tobacco Related Variables**		

Serum cotinine in ng/ml., mean [SD]	275.8 (155.8)	536

Exhaled carbon monoxide in p.p.m., mean [SD]	16.4 (10.0)	411

Cigarettes per day, mean [SD]	8.0 (2.5)	540

Time to first cigarette, ≤30 minutes, n (%)	390 (72.2)	540

FTND, mean [SD]	3.2 (1.7)	540
Smoke menthol cigarettes, n (%)	452 (83.7)	540

Number of previous 24 hour quit attempts in the past year, mean [SD]	3.7 (7.7)	540

Use of pharmacotherapy during the most recent quit attempt, n (%)	133 (24.2)	511

Age of first cigarette, mean [SD] yr	17.6 (5.9)	538

Age started smoking regularly, mean [SD]	21.1 (7.1)	538

Motivation to quit, mean [SD]	9.7 (0.8)	540

Confidence to quit, mean [SD]	7.9 (2.4)	540

Smoke-free household, n (%)	132 (24.4)	540

Retention of participants for Weeks 1, 3, 5, 7, 16, and 26 was 90.6%, 78.7%, 75.7%, 72.8%, 67.6%, and 70.2%, respectively. Of six total counseling sessions, participants completed a mean (SD) of 4.85 (1.49) sessions.

## Discussion

The Kick It at Swope III (KIS-III) trial was designed as the first treatment study of bupropion for African American light smokers. To date, KIS-III is the fourth study to evaluate pharmacotherapy for light smokers [22,77,78] and the second to evaluate treatment for African American light smokers [22]. KIS-III is also the first study to examine both nicotine and bupropion metabolism, evaluating CYP2A6 and CYP2B6 phenotype and genotype as possible predictors of outcome, within the context of treatment of African American smokers. Collecting data on metabolism and genotype allows us to evaluate biological mechanisms in conjunction with psychosocial and smoking data to further advance our understanding of African American smoking behavior and ultimately enhance treatment outcomes.

We have demonstrated the feasibility of recruiting and enrolling African American light smokers into a clinical treatment trial involving biological data collection and genetic analysis. These feasibility findings are consistent with studies that have successfully enrolled African American smokers for treatment and provide further evidence of willingness to share genetic information within a research study of smoking behavior [19,79]. KIS-II enrolled smokers into a cessation trial but conducted the genetic portion of the study using a second consent process. In KIS-II, there was 83% participation in the genetic portion of the study [79]. Based in part on this excellent consent rate, indicating consent for genetic analysis posed little barrier to enrollment, KIS-III included genetic analysis as part of overall study participation and enrolled accordingly. Participants were informed during screening about the goals of the study, the requirement of multiple collections of biological samples, and the rationale for collecting such data. This information was reviewed in further detail within the consent process. Of 1629 individuals who completed any screening for study participation, only 18 (1.1%) were ineligible to participate in the study because they refused blood draws, suggesting that the inclusion of phenotype and genotype assessments did not impede recruitment in this African American population. Factors that contributed to recruitment success may include conducting this research within a trusted community medical setting, having an established history of smoking cessation treatment and research within the community, and using a multi-method approach to recruitment. The current data add to the literature demonstrating support for inclusion of African American smokers in smoking cessation treatment studies, in genetic investigation of tobacco use and treatment, and more broadly in cancer control research.

Consistent with our previous KIS trials, study participants were largely low income and predominantly menthol cigarette smokers. Despite excluding individuals currently using pharmacotherapy for the treatment of depression, we found approximately one third of participants reported symptoms of depression. As other research has shown bupropion to reduce negative mood during smoking cessation treatment [80,81] and to be effective in supporting abstinence in moderate to heavy smokers with or without a history of depression [82], we are planning analyses examining changes in mood and depression in relation to smoking behavior within the current sample of African American light smokers. Importantly, while low income, use of menthol, and depression previously have been associated with poorer smoking cessation treatment outcomes [21,83-87], this sample was highly motivated to stop smoking. Our previous KIS-II trial found health education counseling contributed to higher rates of abstinence in highly motivated African American light smokers, when compared to motivational counseling [22]. The finding that smokers in the current trial show similarly high levels of motivation provided support for the use of health education counseling within the current study.

Examination of KIS-III screening data points to some limitations related to generalizability and recommendations for needed research. Over half of individuals who expressed interest in enrolling in this smoking cessation study were ineligible to use bupropion according to study protocol. Future research should examine other pharmacotherapy for light smokers, such as varenicline, which has been found to be effective in moderate to heavy smokers (>10cpd) [18]. Findings also showed over one third of interested smokers screened in KIS-III reported use of other forms of tobacco and were excluded from treatment. While smoking cessation clinical trials typically exclude smokers who report use of other forms of tobacco, future studies may provide greater generalizability by examining tobacco use treatment more broadly defined. Inclusion of smokers who use other forms of tobacco would require consideration of appropriate measures of tobacco use and abstinence verification.

In summary, the KIS-III study provides a unique opportunity to evaluate psychosocial and biological mechanisms related to drug use and treatment outcomes in African-American light smokers. Baseline findings support the feasibility of enrolling African American light smokers in a treatment study involving genetic analysis. Identifying individuals for whom treatments are most effective and, in contrast, identify characteristics related to relapse risk will contribute to advancing tailored tobacco use treatment, with the ultimate goal of enhancing treatment and reducing tobacco-related health disparities for African-American smokers.

## Competing interests

Dr. Ahluwalia serves as a consultant to Pfizer Pharmaceuticals, Inc. Dr. Benowitz serves as a consultant to Pfizer Pharmaceuticals, Inc. and has been a paid expert witness in litigation against tobacco companies. Dr. Tyndale holds shares in Nicogen Research Inc., a company that is focused on novel smoking cessation treatment approaches: no Nicogen funds were used in this work.

## Authors' contributions

LSC, MSM, NLN, WSC, KSO, NLB, RFT, JSA contributed to the concept and design, acquisition of data, interpretation of data, and drafting of manuscript. BF, RB, and TSS, contributed to acquisition of data, data analysis, interpretation of data and drafting of manuscript. CAB and GAS contributed to acquisition of data. All authors read and approved the final manuscript.
